# Analysis of hand synergies in healthy subjects during bimanual manipulation of various objects

**DOI:** 10.1186/1743-0003-11-113

**Published:** 2014-07-30

**Authors:** Nathanaël Jarrassé, Adriano Tacilo Ribeiro, Anis Sahbani, Wael Bachta, Agnes Roby-Brami

**Affiliations:** 1 CNRS, UMR 7222, ISIR, F-75005 Paris, France; 2 Sorbonne Universités, UPMC Univ Paris 06, UMR 7222, F-75005 Paris, France; 3 INSERM, U1150, Agathe-ISIR, F-75005 Paris, France

**Keywords:** Postural synergies, Kinematics, Fingers, Human

## Abstract

**Background:**

Hand synergies have been extensively studied over the last few decades. Objectives of such research are numerous. In neuroscience, the aim is to improve the understanding of motor control and its ability to reduce the control dimensionality. In applied research fields like robotics the aim is to build biomimetic hand structures, or in prosthetics to design more performant underactuated replacement hands. Nevertheless, most of the synergy schemes identified to this day have been obtained from grasping experiments performed with one single (generally dominant) hand to objects placed in a given position and orientation in space. Aiming at identifying more generic synergies, we conducted similar experiments on postural synergy identification during bimanual manipulation of various objects in order to avoid the factors due to the extrinsic spatial position of the objects.

**Methods:**

Ten healthy naive subjects were asked to perform a selected “grasp-give-receive” task with both hands using 9 objects. Subjects were wearing Cyberglove ^Ⓒ^ on both hands, allowing a measurement of the joint posture (15 degrees of freedom) of each hand. Postural synergies were then evaluated through Principal Component Analysis (PCA). Matches between the identified Principal Components and the human hand joints were analyzed thanks to the correlation matrix. Finally, statistical analysis was performed on the data in order to evaluate the effect of some specific variables on the hand synergies: object shape, hand side (i.e., laterality) and role (giving or receiving hand).

**Results:**

Results on PCs are consistent with previous literature showing that a few principal components might be sufficient to describe a large variety of different grasps. Nevertheless some simple and strong correlations between PCs and clearly identified sets of hand joints were obtained in this study. In addition, these groupings of DoF corresponds to well-defined anatomo-functional finger joints according to muscle groups. Moreover, despite our protocol encouraging symmetric grasping, some right-left side differences were observed.

**Conclusion:**

The set of identified synergies presented here should be more representative of hand synergies in general since they are based on both hands motion. Preliminary results, that should be deepened, also highlight the influence of hand dominance and side. Thanks to their strong correlation with anatomo-functional joints, these synergies could therefore be used to design underactuated robotics hands.

## Background

The building of synergies - that are also usually called inter-joint coordination- is a fundamental aspect of human motor control to overcome the redundancy of the motor system, as pointed originally by N. Bernstein [1]: synergies are solutions to the problem of selecting one movement among the infinite possibilities of motor solutions to perform a specific task. Mathematically speaking, synergies can be represented as task specific covariations of elemental variables with the purpose to stabilize a performance variable [2]. A synergy can be expressed in several spaces: muscular activities, joint positions, joint velocities or joint torques. A recent view attributes three essential properties to the concept of synergy: the sharing pattern of rotations; flexibility allowing automatic compensation between elements and task dependency [2].

Several research groups have already been working on the identification of hand synergies, especially aiming at designing under-actuated hand prosthetics. Santello et al. in [3] studied postural synergies in healthy subjects grasping objects. Experiments were conducted during which subjects were asked to realize hand postures mimicking the grasp of 57 objects, instead of grasping them physically. Two principal components (PC) explained more than 80% of the variance observed among the 15 measured hand DoFs (Degrees of Freedom): the first PC represented the coupling of flexion/extension of metacarpal-phalangeal (MCP) and abductions joints whereas the second PC represented the thumb rotation and the flexion/extension of the inter-phalangeal joints.

E. Todorov and Z. Ghahramani [4] presented a synthesis of results obtained from the study of hand synergies during the manipulation of different kind of objects. Their approach relied on the use of Principal Component Analysis (PCA) and generated similar results: hand grasping postures can be accurately represented with a reduced number of components.

Vinjamuri et al. [5] presented a classical evaluation of hand postural synergies based on the use of the Singular Value Decomposition (SVD, i.e. a matrix factorization) method. Their study showed that the two first components they identified explained 82% of the hand postures.

Further studies of reaching to grasp virtual or real objects showed that the synergies were built during the reaching movement [6,7]. Once again two PCs explained 70% of the variance during reaching but a greater number of PCs was needed for grasping, particularly with real objects. The difference may be induced by mechanical constraints due to the contact and/or to the somatosensory feedback. The lower order PCs coded for the general shape of the hand (opening-closing) when the higher order PCs coded for the more subtle characteristics of objects. Together these studies show that the dimensionality of the kinematic space is smaller than that defined by the number of hand’s mechanical DoFs [8]. The coupling between DoFs of the hand is also observed in kinetic tasks implying the control of contact forces between the fingers and object [9]. Some coupling between fingers and more generally DoF is also observed in tasks were the instruction is to perform individual finger movements (such as piano playing [10]) of force production in which the influence of the activation of one finger on the others is called the enslaving effect [11].

As can be seen from the research works listed above, hand synergies have been extensively studied over the last few decades. Nevertheless, most of the synergies schemes identified to this day have been obtained from manipulation experiments performed with one single (generally dominant) hand. In a recent study [12], we demonstrated that the configuration of the hand and fingers at the time of grasping was influenced not only by the shape and size of the objects but also by their spatial positions. So, in the present study we used a bimanual experimental paradigm with a giving and a receiving hand in order to identify synergy schemes influenced by anatomo-functional characteristics of the human hand rather than by extrinsic spatial position of the objects. Due to this bimanual experimental design, we also had to consider higher-level control phenomena like laterality and the role (giving or receiving) of the hand.

## Material and methods

### Model of the human hand

The human hand kinematics has 28 DoF. Each finger has 4 DoF: 2 flexion/extension mobilities between phalanxes (Proximal Inter-Phalangeal hinge joints (PIP) and Distal Inter-Phalangeal hinge joints (DIP)) along with 2 DoF at the MetaCarpal Phalangeal (MCP) saddle joint (flexion-extension and abduction/adduction mobilities). The thumb has 5 DoF: 2 Flexion-extension mobilities thanks to the Proximal Inter-Phalangeal and MetaCarpal-Phalangeal hinge joints and at least 2 DoF at the level of the saddle joint between the carpus and metacarpus (trapeziometacarpal joint). In addition to these mobilities, the thumb exhibits a pseudo-rotation allowing 3 DoF. Extra DoF of the hand are located at the wrist, between the carpal bones, to modify the palmar arch concavity. As most precision and intermediate grasps are mainly performed by the finger and thumb [13], we voluntarily simplified the hand model by not considering the palm and wrist joints.

**Figure 1:**
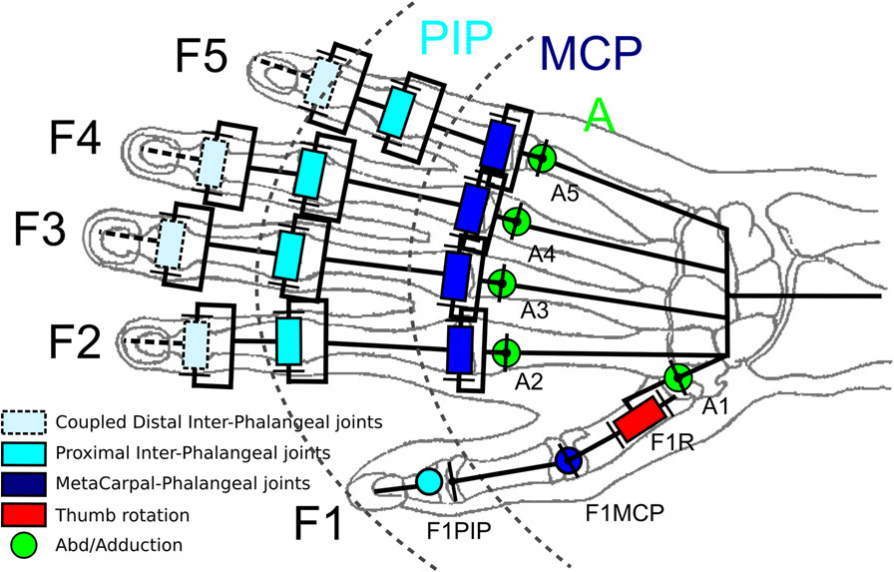
**Schema of the 15 DoF kinematic model considered.** All these joints were measured during the experiments. Nevertheless, because of the glove technology, “coupled abductions” of the fingers (i.e., abduction angle between fingers) were recorded (A12, A23, A34, A45) instead of individual abduction (A1, A2, A4, A4, A5) of each finger. F*x*PIP stands for finger *x* Proximal Inter-Phalangeal joints and F*x*MP for finger *x* Metacarpal-Phalangeal joints. The Distal Inter-Phalangeal joints are not considered as they are coupled with and proportional to the Proximal Inter-Phalangeal (PIP) joints.

The hand kinematic model used for our experiments is shown on Figure 1. Although the figure shows 20 DoF, only 15 joints are actually recorded by the Cyberglove. First the Distal Inter-Phalangeal (DIP) of the gloves are not fitted with sensors, and therefore were not considered in this study. In addition, they are mechanically coupled (through tendons) to the Proximal Inter-Phalangeal (PIP) joints. Secondly, since the Cyberglove technology relies on flexible sensors placed between fingers for abduction sensing, it does not measure the five individual finger rotation (A1, A2, A3, A4, A5) but four “coupled abductions” (A12, A23, A34, A45), i.e. the angles between fingers rotating during abduction movements.

The following codification was therefore used (see Figure 1): 

•F1, F2, F3, F4 and F5, respectively identify the thumb, the index, the major, the ring and the pinkie;

•MCP and PIP, respectively represent the MetaCarpal-Phalangeal joints and the Proximal Inter-Phalangeal joints of the fingers;

•A (*i*,*i*+1) (with *i*∈{1,..,4}) represents the abduction and adduction joint between a finger *i* and the following one (*i*+1);

•F1R represents the thumb rotation responsible for the opposition gesture.

### Cyberglove and calibration

For our study, two Cyberglove ^Ⓒ^ II (18 sensors gloves among which only 15 were used) were used to record the thumb and the fingers joint values with a precision below one degree at a frequency of 100 Hz. Such devices rely on the use of piezoelectric sensors which are sewn inside the elastic glove at each joint level. Bending of one sensor generates a variation of its output voltage, proportionally to the angular posture of the joint. However, even if the flexible glove can adapt to many hand sizes, it has to be calibrated for each subject in order to get representatives measurements independent of the subject’s hand morphology.

**Figure 2:**
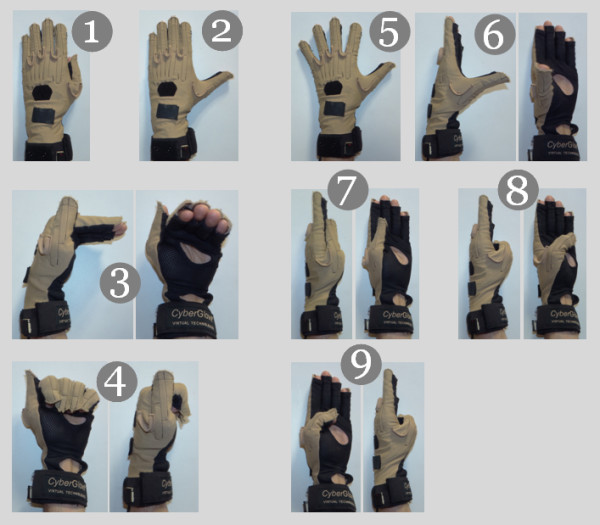
**Key-postures that every subject had to perform once during the calibration phase.** This phase was performed once by each subject prior to the grasping tasks and the calibration obtained was then used during the entire experimental session.

We therefore developed a simple and fast calibration method derived from the procedure introduced in [14] which relies on the classical use of key-postures. A specific effort has been made to define these key-postures. During the calibration phase, each subject wearing the glove is then asked to reproduce a certain number of known and defined postural patterns with their hand. These patterns are chosen in order to explore a large part of the joint space of the human hand (see Figure 2). As the theoretical angular values for this group of postures are known, a simple linear regression is then performed on the experimentally recorded value in order to identify the gain and offset values (for each sensor) to apply to the raw data sent by the Cyberglove ^Ⓒ^ in order to get accurate angular measurements during the experiments:

(1) $$\alpha =\mathrm{G.}(D_{b}-O_{\text{ff}})$$

in which *α* is the angular value in degrees, *D*_*b*_ the raw data, *G* the gain factor and *O*_*ff*_ the offset are the two identified values. An optimal estimation through a least square minimization is performed over the set of recorded postures to identify these parameters. During our experiments, calibration was performed once by each subject before the grasping task and the calibration data were kept and used during the entire session since the subjects did not remove the glove during this time.

### Experimental protocol

Ten healthy subjects (22–28 years old, 8 men and 2 women, among which 8 right-handed by self report) naive to the experiment, volunteered to participate to this study. All participants were self-reported to be in good health, with no history of neurological or motor disorders. The present study in healthy subjects is a preliminary control experiment in the framework of a protocol on analysis of dexterity in patients reviewed and approved by a local ethics committee, the “Conseil d’Évaluation Éthique pour les Recherches en Santé” of University Paris Descartes. All participants provided informed consent before the experiments, as required by the Declaration of Helsinki.

**Figure 3:**
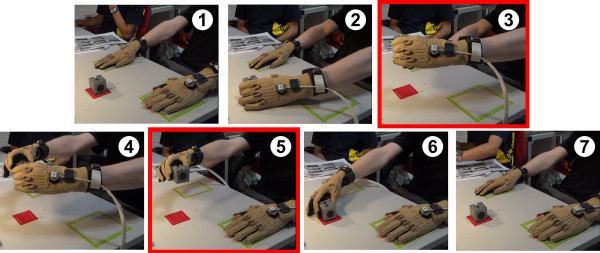
**Illustration of the different protocol’s steps for a subject starting with his left hand.** Hand’s start areas are delimited with yellow/green tape and object’s area with red tape. First subject places both his hands on the start areas **(1)**, then he/she is asked to grasp the object with the selected starting hand and to lift it **(2)**, to hold it still **(3)**, then to transfer the object to the other “receiving” hand **(4)**, to hold the receiving hand still **(5)**, then finally to release the object **(6)** and to return to the start areas **(7)**.

Each subject was asked to “grasp-give-receive” 9 different objects, twice. These objects were chosen specifically to allow different grasp types and to span most of hand workspace (see Table 1). The following protocol has been adopted for each grasp-give-receive task (see Figure 3). The subject had to: 

1. Place both his hands (wearing Cybergloves) on the start areas delimited by yellow/green tape.

2. Grasp the object in a natural way with the selected starting hand and lift it.

3. Hold still for 2 seconds.

4. Transfer the object to the other “receiving” hand.

5. Hold his/her “receiving” hand still for 2 extra seconds while returning his/her giving hand to the start area.

6. To release the object in the start area.

7. To return his/her “receiving” hand to its start area.

**Table 1:** Intrinsic properties of the nine objects

**Object**	**Mass in g**	**Grasping dimensions in mm**
Mug’s handle	320	Circular handle diameter 60 mm,
		section diameter 14 mm
Tape roll	460	Diameter: 110 mm; height:50 mm
Handle	140	Cylinder length: 100 mm
		section diameter 30 mm
Cube	170	Side: 45 mm
Box	340	Height: 108 mm
Ball	20	Diameter: 75 mm
Nut (M16)	12	Diameter: 19 mm; height: 11 mm
Thin box	125	Thickness: 17 mm
Pen	8	Diameter: 9 mm; length:130 mm

**Figure 4:**
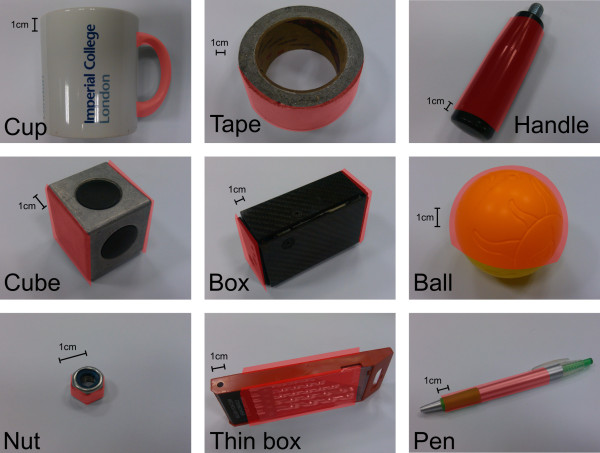
**The nine objects used for the experiments: the handle of a mug, a large tape toll, a handle, a 4 cm metallic cube, a 300 grams parallelepiped box, a hollow plastic ball, a M12 nut, a thin rectangular drill bits box and finally a ballpen.** The experimenter instructed the subject to grasp the areas of interest highlighted in red.

The “grasp-give-receive” task allows us to obtain more generic synergies since both hands are involved. This lowers the influence of hand dominance and side. The selected objects were placed in the object area (delimited by red tape) presented to the subject by the experimenter. Since some objects could be grasped in different ways, some areas of interest for the grasping or affordances (ie, “the design aspect of an object which suggest how the object should be used; a visual clue to its function and use” [15], for example the handle area for the cup [16]) were indicated to the subjects. These areas of interest are shown on highlighted red areas in Figure 4). The objective was to generalize results among the group by avoiding unconventional grasping strategies by uncommon areas. Despite this indication, an important inter-subject variability was observed.

Starting hand (among right and left) was chosen randomly across subjects and then kept as starting hand for all the trials. The whole procedure lasted 20 minutes.

### Data analysis and statistics

**Figure 5:**
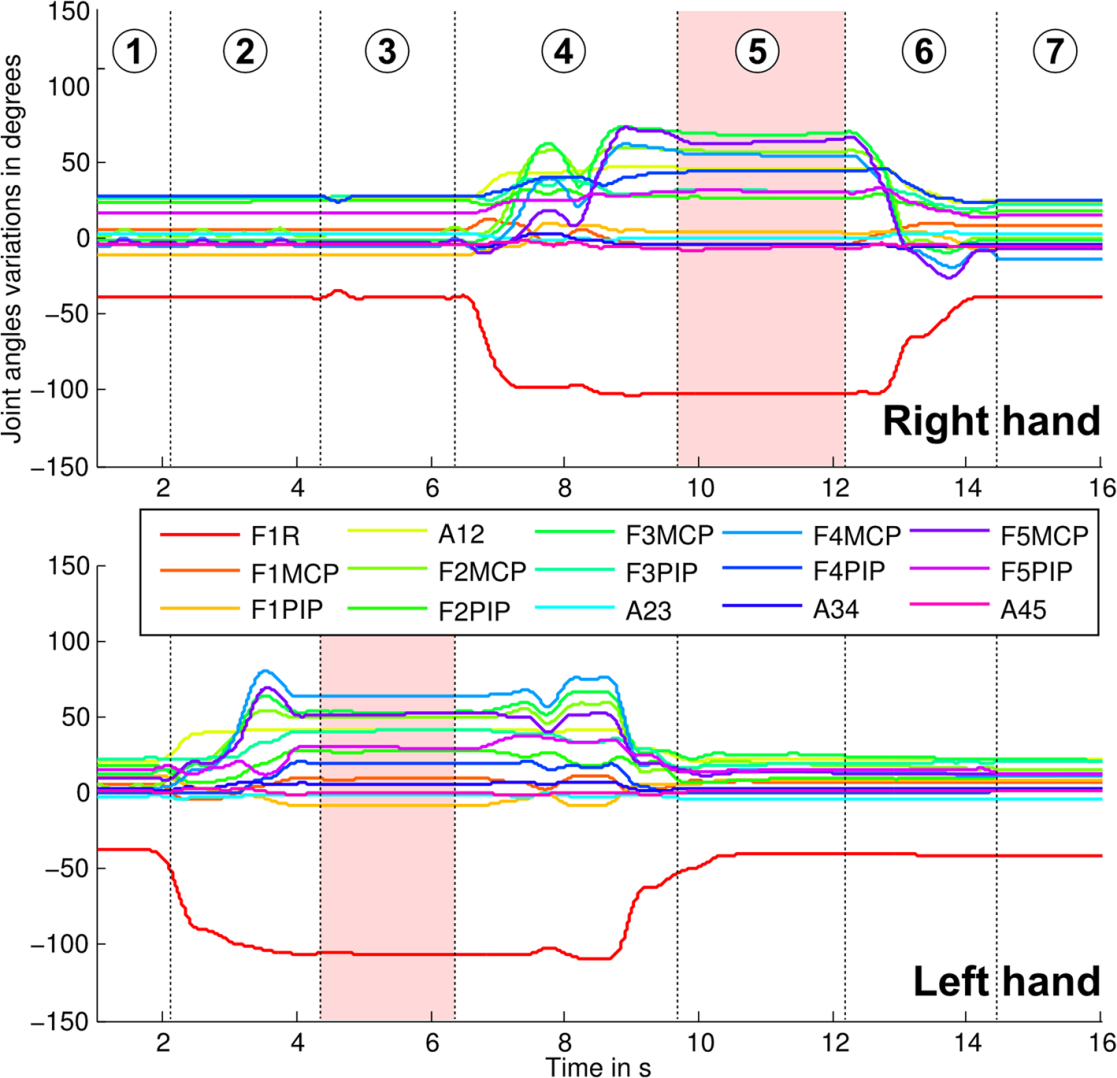
**Example of recording of the 15 Dof of both hands during the grasping of the nut.** The 7 phases described Figure 3 are indicated. Hand postures for PCA calculations are averaged over the two red areas. F*x*PIP stands for finger *x* Proximal Inter-Phalangeal joints and F*x*MP for finger *x* Metacarpal-Phalangeal joints, A*xy* for abdution between fingers F*x* and F*y*, and F1R for the thumb rotation.

Trajectories recorded for every joint were filtered (low-pass filter with a 10 Hz cutoff frequency) in order to remove noise. An example of recorded trajectories during the grasping of an object can be seen in Figure 5 in which one subject is grasping the nut with his left hand first, then with his right hand.

Grasping postures for both “giving” and “receiving” hands were extracted from the recordings (steps highlighted in red in Figure 3 equivalent to the transparent red areas from Figure 5) and averaged over the two trials in order to perform some postural synergies evaluation through Principal Component Analysis (PCA).

Principal Component Analysis or PCA is a technique from statistics for simplifying a data set that was developed by Pearson [17] and Hotelling [18]. It aims at reducing the dimensionality of multivariate data whilst preserving as much of the relevant information as possible and can be seen as an orthogonal linear transformation from one coordinate system to another. Mathematically, the transformation is defined by a set of *p*-dimensional vectors of weights or loadings *W*_(*k*)_=(*w*_1_,*w*_2_,…,*w*_*p*_)_(*k*)_ that map each row vector *X*_(*i*)_ of *X* to a new vector of principal component scores *t*_(*i*)_=(*t*_1_,*t*_2_,…,*t*_*p*_)_(*i*)_, given by *t*_*k*(*i*)_=*x*_(*i*)_.*w*_(*k*)_ in such a way that the individual variables of *t* considered over the data set successively inherit the maximum possible variance from *x*, with each loading vector *w* constrained to be a unit vector [19]. PCA were classically calculated with the covariance method. PCA was conducted over all ten subjects, hands (left hand postures were projected on the right side in order to analyze both hands together) and all objects. The PCA data consisted of two sets of [180 × 15] matrix (i.e. 10 subjects × 9 objects and 15 kinematic DoFs for both hands).

We used the Joliffe criterion [19] (based on the analysis of the eigenvectors combined inertia and the definition of a reference threshold) to limit the number of considered eigenvectors. In our analysis, the threshold was set to 0.95 (in order to cumulate 95% of the energy with the eigenvectors).

Correlation analysis was performed between each of the 15 DoFs and the PCs scores. Two-Factor ANOVA with Repeated Measures were performed (Statview ^Ⓒ^ software) to evaluate the influence of the hand side and role, and object shape. The dependent measures were the PCs scores split according to the condition. The independent Factors were Object (9 levels) and Hand (2 levels) with the subjects as repeated measures. Since the subjects randomly used the left or right hand for giving or receiving, the analysis was performed twice: with the factor Hand indicating right-left side (Side) and with the factor Hand indicating the giving-receiving role (Role).

## Results

### Demonstration of synergies between DoFs

Principal component analysis showed a clear reduction of the variable’s space dimension since the first 4 PC are representing more than 95% of the postural variability

**Figure 6:**
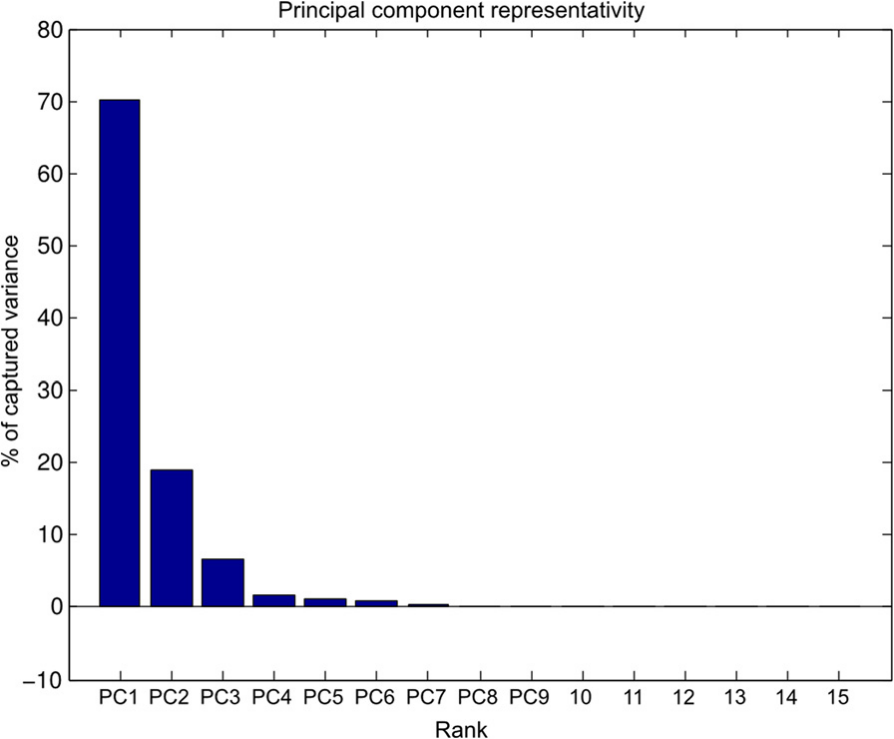
**Percentage of representation of each of the principal components calculated for both hands (left hand projected on the right one).** A clear reduction of the variable’s space dimension can be observed, as the 4 first PC are representing more than 95% of the postural variability.

As shown on Figure 6 which illustrates the percentage of variance explained by each component, the first PC (PC1) codes for 70% of the grasping postures, the second one PC2 for 18%, third one PC3 for 5% and finally PC4 for 3%.

### Mapping of the PCs on the DoFs

**Figure 7:**
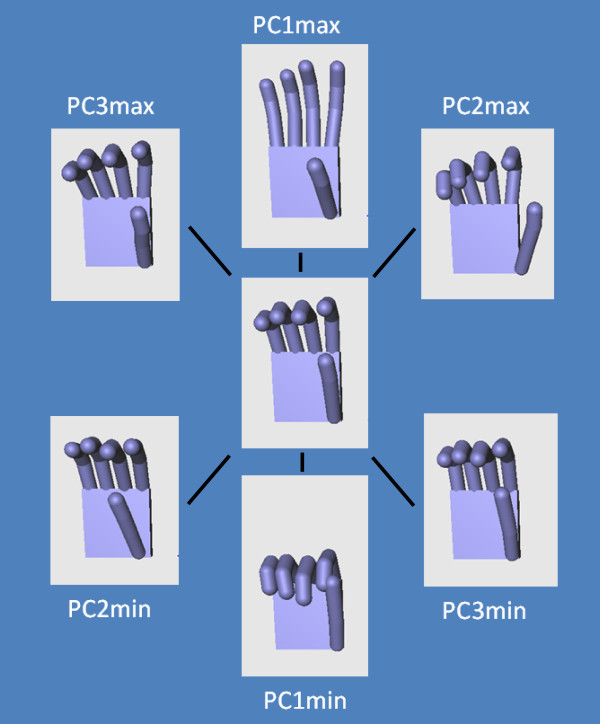
Visualization of the min and max postures of the 3 first principal components of the grasping postures obtained with the C++/OpenGL software that was developed for the experiments.

The mapping of the PCs on the DoF of the hand is illustrated on Figure 7 which displays the min and max values of the 3 first identified principal components. This mapping is further quantified by the correlation matrix presented in Table 2 which precisely identifies the matches between the identified Principal Components and the human hand joints.

**Table 2:** Detail of the correlation matrix

	**PC1**	**PC2**	**PC3**	**PC4**
**F1MCP**	0.023	0.167	**0.613**	0.075
**F1PIP**	-0.345	0.142	-0.178	**0.61**
**F1R**	0.021	-0.108	-0.344	**-0.786**
**A12**	0.202	-0.231	-0.148	**0.69**
**F2MCP**	-0.006	**0.819**	0.18	-0.009
**F2PIP**	**0.756**	0.14	-0.164	0.099
**A23**	-0.032	-0.178	**-0.713**	0.115
**F3MCP**	0.087	**0.849**	0.16	-0.0004
**F3PIP**	**0.815**	-0.026	-0.086	-0.032
**A34**	-0.155	-0.144	**0.754**	-0.168
**F4MCP**	0.007	**0.896**	-0.0002	0.01
**F4PIP**	**0.874**	-0.084	0.059	0.013
**A45**	0.09	-0.179	**0.77**	0.169
**F5MCP**	-0.065	**0.891**	-0.338	-0.041
**F5PIP**	**0.824**	0.048	0.138	0.039

The analysis of the matrix shows (see Table 2) strong correlations between PCs and joints (highlighted in bold): PC1 is strongly correlated with the Proximal Inter-Phalangeal joints (PIP); PC2 with the MetaCarpal-Phalangeal joints (MCP); PC3 gathers fingers coupled abductions (A (*i*,*i*+1)) and F1MCP, and finally PC4 with the thumb joints (F1).

### Influence of object and side factors on the synergies

**Figure 8:**
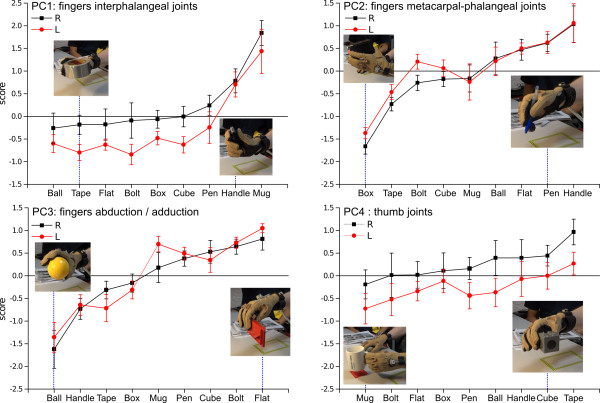
**Scores of PC1 to PC4 for the grasping of the nine objects by each hand.** The figure presents the scores for each PC split according to the object grasped. The objects are ranked according to the amount of the corresponding PC score and the photographs illustrate representative finger configurations for the minimum and maximum scores. The right (Black squares) and left (red circles) hands are represented separately.

There was a highly significant effect of the Factor “object” on the PCs scores which was statistically verified for the first three PCs ($F_{72}^{8}=12.680$, *p*<0.0001 for PC1, $F_{72}^{8}=16.449$, *p*<0.0001 for PC2 and $F_{72}^{8}=22.007$, *p*<0.0001 for PC3). Figure 8 shows the variation of the scores of each PC with the grasped object and with the Side.

The PC1 representing the interphalangeal joints is naturally highly explored when the subject is grasping thin objects around which the fingers are bending such as the pen (grasped by subjects in the way they would hold it for drawing or writing), the handle or the mug (grasped by the handle); and weakly used to grasp large size objects requiring important finger extension (the ball or the large scotch roll). The PC2 representing the metacarpal-phalangeal joints are involved in the grasping of thin or revolution objects with a small radius, like the pen, the handle or the flat drill bits box. Such objects tend to be grasped with a multiple finger precision grip (thumb facing other extended fingers). These joints are obviously less involved in the grasping of the large objects (like the box object). The PC3 coding for the adbuction/adduction naturally shows a larger spread of fingers to grasp large objects (small values of PC3) like the ball, the handle or the large tape roll; and bigger adduction to grasp smaller objects like the thin drill bits box or the nut.

The PC4 relative to the object is more difficult to analyze (see Figure 8) as it combines some flexions of the phalangeal joints and the rotation of the thumb. Therefore different grasping postures with important thumb rotation (power grasp of the cube) or thumb extension (the grasping posture of the large tape roll for example) will generate similar large values for PC4.

The effect of the hand side was also statistically significant for PC1 ($F_{72}^{1}=9.265$, *p*<0.01) and particularly for PC4 ($F_{72}^{1}=28.212$, *p*<0.0005). In contrast, there were no statistically significant effect of the “giving” or “receiving” Role of the hand over the grasping synergies represented by the PCs ($F_{12}^{1}=0.6$, NS).

## Discussion

### Number of PCs and amount of variability explained

Results on PCs are consistent with previous literature results showing that there is an important reduction in the number of degrees of freedom of the hand; and therefore that a few principal components might be sufficient to describe a large variety of different grasps. Similarly to [3], two principal components are able to account for more than 80% of the variance in the data.

### Anatomical identification of the PCs

The originality of the present study is that it evidences some simple and strong correlations between PCs and clearly identified sets of hand joints.

The striking observation is that the 4 PCs emerging from our data are strongly correlated with specific hand DoF. In addition the grouping of DoF corresponds to well-defined anatomo-functional muscle groups. PC1 is correlated to the Proximal Inter-Phalangeal Joint (PIP) of the four fingers. It is well known that the flexion of the distal phalanx is due to extrinsic finger flexor muscles which are situated in the forearm, their tendon travelling through the carpal tunnel to attach to the intermediate (superficialis) or to the distal (profundus) phalanx of the four fingers. Similarly, extension of the distal phalanges is due to the extrinsic common extensor muscle (extensor digitorum). In contrast, the flexion of the MetaCarpo-Phalangeal (MCP) joints, with extended interphalangeal joints, corresponding to PC2, is likely due to the intrinsic (with their origin and insertions in the hand) lumbrical muscles. PC3 is correlated with abduction of the fingers and with the DoF of the thumb moving it away from the main hand axis. This movement is due to the activation of the dorsal interossei muscles. PC4 is correlated to three DoF of the thumb. The mobility of the thumb is due to both extrinsic (e.g. flexor pollicis longus) and intrinsic (e.g. opponens pollici) muscles. The biomechanics of the thumb and particularly the number of anatomical DoF of the trapezometacarpal joint remains disputed [20,21]. Opposition may involve axial rotation of the metacarpal bone but this movement cannot be measured with the dataglove. Further experimental and theoretical studies are clearly needed to precise this point.

Such a functional meaning of each PC was not clearly reported before. Rather, previous study suggested that the global pattern of hand muscle activation is highly distributed: multiple EMG recordings showed that there is no direct relationship between each muscle and a given hand shape [22]. The good functional association of PCs observed here may be due to our particular task, since we recorded real hand-object interactions and since the object was grasped thanks to a bimanual transfer and not fetched by a reach to grasp movement. The bimanual transfer situation avoids the contamination of the grasping posture by the potential effect of object position or orientation which may influence hand and finger configuration for grasping [12]. Such preliminary experiments should still be reproduced on a larger population of subject to rigorously validate the results that are shown in this paper.

### Mechanisms of coupling

The first PC corresponds to a global opening-closing of the four fingers of the hand, consistently with previous studies [7,23,24]. The coupling between flexion of the four fingers may be due to the fact the flexor muscles raise 4 tendons and which are mechanically coupled in the carpal tunnel and by a rich fibrous aponeurosis and tendon web in the palm of the hand. So, the activation of one motor unit corresponding to one finger may biomechanically spread to other fingers [25]. However the coupling between fingers is more likely due to neurophysiological factors. Multi-digit muscular motor units have been discovered in monkeys, but not in man [26]. The main cause of coupling between finger flexors is probably central [11,27]. At the cortical level, the representation of the different fingers is largely overlapping, with both convergence (large cortical areas activating one finger) and divergence (a same cortical zone activating several fingers) [28]. The common cortical drive synchronizes the discharge of finger flexor motoneurons [29]. In addition, coupling between fingers and synergies could be due to an intermediate sub cortical level of control at spinal level [30,31].

As proposed by Santello [23], the lower range synergies could refine the control of the hand shaping by a fine adaptation to the shape of the object. Our observations are consistent with this view since PC2 and PC3 may contribute to adjust the global curvature of the hand shape in two different planes orthogonal to the palm. The synergic control of PC2 on the MCP joint is consistent with the observation that coupling between fingers can also be observed at this level [11]. Contrary to extrinsic muscles, intrinsic muscles are not anatomically coupled, but enslavement effects are similar whether the force is applied at the first phalanx or at the tip of finger, suggesting that coupling between fingers is mainly central.

PC3 controls the abduction of the fingers and F1MCP and may adapt the hand shape to the width of different objects. In the anatomical reference posture, finger abduction is performed in the plane of the palm and finger flexion in a plane perpendicular to the palm. This distribution (gathering of F1MCP and A (*i*,*i*+1) joints) makes sense when considering that, in functional hand postures, the thumb column is generally directed in front of the palm and internally rotated so that flexion in F1MCP is performed in a plane roughly parallel to the palm. In that sense F1MCP extension contributes to hand span together with the abduction of the fingers.

In contrast, PC4 gathers the three other thumb DoF (A12, F1R and F2PIP). A12 and F1R quantify the coupling in the trapezometacarpal joint leading to thumb orientation toward the palm and finger tips for opposition, that can be finalized by F2PIP. PC4 was mainly related to a combined motion of the thumb, probably implying an association of flexion and axial rotation or opposition. This segregation of thumb flexion from other fingers’ flexion may appear surprising since some coupling has been described in force production tasks [25]. However, PC4 may have a strong functional meaning by coding the site of opposition of the thumb against e.g. the index versus the little finger and thus be related to precision grip.

Nevertheless, we have to remind that, firstly, PC4 is only coding for less than 4% of the captured variance and secondly, the thumb rotation is the hardest joint to measure (and to calibrate) with the Cyberglove acquisition system.

In brief, many intricate factors probably intervene to explain the synergistic coupling of DoF for grasping. The osseous anatomy of the hand (model) and other strong biomechanical constraints linked to tendon and fascia, muscle repartition and peripheral innervations are quite important factors. The compliance of the hand apparatus may also contribute to the fine adaptation of the hand to the object [32] and to the increase of the limits [27]. Finally, the central command of hand movements both at spinal and cerebral level are of primary importance for the coupling [27]. It is likely that the coupling emerging from peripheral low level constraints may be learned due to daily use of the hands thanks to brain plasticity [8,27].

### Toward a simplified underactuated robotic hand

Most of the study on postural synergies usually observed rather complex correlations between measured synergies (i.e. PCs) and human joints. Such complex correlation could be easily reproduced with fully actuated robotic hands with numerous DoF (like the DLR hand [33]) by reproducing the synergistic activation at the control level as in [34]. Nevertheless, using a 19 actuators robotic structure to exhibit only a reduced number of degrees of freedom is a waste in terms of cost, complexity, autonomy and extra embedded weight. Reproducing the dimensionality reduction at the mechanical level rather than at the control one could be a better alternative, as long as the bio-inspired correlations (obtained through these studies of human hand synergies during grasping and manipulation) are simple enough to be thought mechanically, thanks to some tendons coupling approaches for example, like in [35] in which a 15 Dof hand is actuated with one single actuator.

An interesting use of this knowledge of hand synergies is shown in [36] in which a 17-degree-of-freedom 5-fingered robot hand is successfully controlled with only two actuators. The proposed hand embed in its mechanical structure hardware synergies (similar to those experimentally identified on human subjects) and a mechanism to switch between sets of eigen-postures. The final hand is complex. Through the strong correlation between PCs and anatomo-functional joints, our results, should induce a simplified mechanism design.

### Investigating the left-right hand synergistic differences

An interesting unexpected result is the statistically significant effect of the hand side while the protocol tended to encourage the subjects to exhibit more postural symmetries between both hands (because of the proximity of the visual reference of the giving hand posture). Such an effect would have been more expected during a typical successive grasp/release task.

Surprisingly, the effect of laterality and handedness on finger kinetic or kinematic synergies [37,38] has been studied very little. A recent study shows no difference between dominant and non-dominant hand for finger enslaving [39]. According to our results however, the side indeed seemed to influence the PC1 and PC4 (respectively coding for the finger interphalangeal joint and the thumb joints). Similar results were observed in [40] without dedicated investigation.

This should be further investigated considering the importance of handedness for human dexterity. While most existing bimanual systems assume symmetry between left and right hands, results presented in this paper invites to reconsider this basic assumption. Further works will investigate the “side” effect during a bimanual task by defining an appropriate protocol whose objective will not accentuate the mirror effect between the two hands, using a larger population of subjects with a more balanced number of left and right handed subjects.

## Competing interests

The authors declare that they have no competing interests.

## Authors’ contributions

ATR carried out experiments under the supervision of AS, NJ, WB and ARB. NJ and ARB analyzed the data and wrote the paper. AS and WB helped in revising the manuscript. All authors read and approved the submitted manuscript.
